# Phylogenetic Analysis and Antimicrobial Profiles of Cultured Emerging Opportunistic Pathogens (Phyla Actinobacteria and Proteobacteria) Identified in Hot Springs

**DOI:** 10.3390/ijerph14091070

**Published:** 2017-09-15

**Authors:** Jocelyn Leonie Jardine, Akebe Luther King Abia, Vuyo Mavumengwana, Eunice Ubomba-Jaswa

**Affiliations:** 1 Department of Biotechnology, University of Johannesburg, Doornfontein, 2094 Johannesburg, South Africa; theworkbodytalk@gmail.com (J.L.J.); vuyom@uj.ac.za (V.M.); 2 AMBIO Environmental Management, Department of Biotechnology, Vaal University of Technology, Andries Potgieter Blvd, Private Bag X021, Vanderbijlpark 1911, South Africa; lutherkinga@yahoo.fr; 3 Water Research Commission, Private Bag X03 Gezina, Pretoria 0031, South Africa

**Keywords:** emerging opportunistic pathogens, hot springs, antibiotic resistance, phylogenetic analysis, Actinobacteria, Proteobacteria

## Abstract

Hot spring water may harbour emerging waterborne opportunistic pathogens that can cause infections in humans. We have investigated the diversity and antimicrobial resistance of culturable emerging and opportunistic bacterial pathogens, in water and sediment of hot springs located in Limpopo, South Africa. Aerobic bacteria were cultured and identified using 16S ribosomal DNA (rDNA) gene sequencing. The presence of *Legionella* spp. was investigated using real-time polymerase chain reaction. Isolates were tested for resistance to ten antibiotics representing six different classes: β-lactam (carbenicillin), aminoglycosides (gentamycin, kanamycin, streptomycin), tetracycline, amphenicols (chloramphenicol, ceftriaxone), sulphonamides (co-trimoxazole) and quinolones (nalidixic acid, norfloxacin). Gram-positive *Kocuria* sp. and *Arthrobacter* sp. and gram-negative *Cupriavidus* sp., *Ralstonia* sp., *Cronobacter* sp., *Tepidimonas* sp., *Hafnia* sp. and *Sphingomonas* sp. were isolated, all recognised as emerging food-borne pathogens. *Legionella* spp. was not detected throughout the study. Isolates of *Kocuria*, *Arthrobacter* and *Hafnia* and an unknown species of the class Gammaproteobacteria were resistant to two antibiotics in different combinations of carbenicillin, ceftriaxone, nalidixic acid and chloramphenicol. *Cronobacter* sp. was sensitive to all ten antibiotics. This study suggests that hot springs are potential reservoirs for emerging opportunistic pathogens, including multiple antibiotic resistant strains, and highlights the presence of unknown populations of emerging and potential waterborne opportunistic pathogens in the environment.

## 1. Introduction

Precious water resources are threatened by pathogen pollution due to human activities, and waterborne diseases are well described and investigated globally, including in South Africa [1,2,3]. Well-known waterborne bacterial pathogens include *Escherichia coli*, *Campylobacter*, *Vibrio cholera*, *Helicobacter*, *Shigella*, *Pseudomonas*, non-tuberculosis mycobacteria and *Legionella*, while protozoa infections also include *Acanthamoeba* and *Naegleria fowleri* [2]. Most waterborne diseases result in diarrhoea, and globally more than 2.2 million children die each year as a result. In South Africa, diarrhoea is the leading cause of mortality in children under five years old [4].

Although hot springs are generally associated with healing practices like balneotherapy or hydrotherapy [5,6], they also carry a potential for infection by microorganisms, as indicated by the isolation of the abovementioned pathogens from swimming pools [7], and thermal baths associated with hot springs [8,9]. The potential of infection appears to be linked to the level of human activity and contact, and these organisms are not the dominant disease-causing microorganisms in the waters of pristine hot springs, i.e., water that has had no previous contact with human or animal activity. Infections from hot springs are rare and sporadic and mostly associated with *Legionella* [10] and free-living amoeba (*Acanthamoeba* and *Naegleria fowleri*) [11]. Other protozoa, such as *Vittaforma* [12] and fungi *Ochroconis gallopava* [13], have also been reported to cause infections associated with hot springs.

The terms “emerging and opportunistic” pathogens require current definition and review since they are described relative to time and the associated vulnerable populations. An emerging human pathogen is defined as the etiological agent of an infectious disease whose incidence has increased in the past 20 years and will probably do so in the future. In several earlier reviews on emerging waterborne pathogens, *Legionella* and *Enterobacter sakazakii* [1] were mentioned. The latter, later renamed *Cronobacter sakazakii*, was isolated in baby formula and is an emerging pathogen of infants and neonates [14,15,16]. On the other hand, opportunistic pathogens are described as pathogens causing infections that exploit opportunities not normally available, such as a host with a weakened immune system, an altered microbiota (such as a disrupted gut flora), or breached integumentary barriers [17]. Pathogens that are both opportunistic and emerging, including *Pseudomonas* and *Ralstonia* [16] *Kocuria* [18,19], *Sphingomonas* [20] and *Cupriavidus* [21], have been described in more recent publications.

Opportunistic pathogens, by their nature, are not clear-cut harmful or harmless, and infections are rather a reflection of the host’s state of health and immunity. Commonly, these bacteria are ubiquitous in the environment and are classified as the lowest level of biohazard, i.e., Level 1 [22]. They can, and do cause infections in debilitated or immunocompromised individuals (including neonates, infants, the elderly, those infected with the human immunodeficiency virus/acquired immune deficiency syndrome (HIV/AIDS) and cystic fibrosis patients) and include 27 genera as listed by Berg et al. [23]. They also commonly cause nosocomial infections [24,25]. Eight percent of acute gastrointestinal infections in the USA are from unknown causes [26], and Dewaal et al. [4] also described the causative agents of a substantial proportion of global diarrheal outbreaks, which remain unspecified. These opportunistic and emerging pathogenic bacteria may be the missing link in waterborne diseases of unknown aetiology. Case studies and outbreaks due to these opportunistic pathogens are listed in Table S1 (Supplementary Materials), showing the diversity of possible infections, global location of infections and risk factors associated with the host’s condition. It is well known that these bacteria, including *C. sakazakii* [13], *Hafnia* [27], *Sphingomonas* [27,28] and *Legionella* [29], can be isolated from environmental samples such as soil and water. *Cupriavidus* sp. has been isolated from drinking water [30], and *Gulbenkiania* sp. from wastewater [31]. Not only do they exist ubiquitously in the environment, but interestingly they are also found in the rhizosphere microbiome, and on or within plants, and play a role in the production of plant growth factors [16,23].

A variety of bacterial species has been isolated from hot springs. Most cultured bacteria reported from hot springs in other studies [32,33,34] have been described as belonging to the phylum Firmicutes, genus Bacillus, due to their endospore-forming ability, thermotolerance and relatively simple nutritional requirements [35]. However, in metagenomic studies, the Gram-negative phylum Proteobacteria is equally predominant in the microbiome as the phylum Firmicutes [36]. In periodically published reports on bacteria isolated from hot springs, three classes of the Proteobacteria, namely Alphaproteobacteria, Betaproteobacteria and Gammaproteobacteria, have been described, including *Hafnia*, *Ralstonia*, *Tepidimonas*, *Sphingomonas* and *Silanimonas* (Figure S1 and Table S1; Supplementary Materials). It appears that these non-spore-forming bacteria are also able to withstand harsh environmental conditions such as extreme temperature, pH, salinity and radioactivity. It has been reported that the following bacteria thrive under extreme conditions: *Ralstonia* [37], *Legionella* [38], *Kocuria* [39,40], *Tepidimonas* [41] and *Cronobacter* [42]. Except for *Legionella* and *Pseudomonas*, there have been no reports associated with infections by the abovementioned bacteria from hot springs.

The impact of emerging and opportunistic pathogens is largely determined by the health and susceptibility of the population utilising the water resources and in developing countries, this includes AIDS/HIV positive individuals. Globally, in 2016 the prevalence of AIDS/HIV was 0.5% with 3.7 million people infected out of a world population of 7.5 billion [43]. The population of South Africa is vulnerable to opportunistic infections for several reasons. In 2016 in South Africa, the prevalence of HIV was 12.7% [44]. Of the 539,714 deaths recorded by the country, 27.9% were HIV-related. Approximately 10.4% of the population is <5 years old, while about 8% is >60 years old. Consequently, 18% of the population fall into the very young and elderly fractions. Malnutrition also weakens the immune system and is a major concern in the country where the growth of one in five children under five years of age is reportedly stunted due to malnutrition [45].

Using bacterial cultivation and culture-independent techniques, the aim of this study was to isolate culturable emerging opportunistic bacteria from hot springs located in Limpopo Province and to determine the role these bacteria may play on water safety and health. Findings were discussed in the context of potential opportunistic infections in a population of individuals who may be immunocompromised for several reasons and who find it necessary to use local groundwater for various domestic activities. Furthermore, since it is estimated that <1% of microbes have been cultured from hot springs [46], any additional information on the diversity of culturable bacteria isolated from hot springs will add to current knowledge.

## 2. Materials and Methods

### 2.1. Sampling Sites

Most of the South African hot springs are located in the Limpopo Province. Five hot springs (Tshipise, Siloam, Mphephu, Lekkerrus and Libertas) were selected based on temperature and ease of sampling.

The geographical and physicochemical characteristics of the hot-water springs have previously been reported by Olivier et al. [47]. Temperature and pH were measured in situ using the YSI Professional Plus Electrode (Xylem Water Systems, Inc., Rye Brook, NY, USA). Dissolved oxygen (DO) was too low to be measured (below the detection limit of the instrument). Table 1 provides the global positioning system (GPS) coordinates and the conditions of the hot-spring sampling sites in Limpopo Province that were sampled.

Except for Siloam, the other hot water springs have all been developed into resorts for human recreational purposes. Water samples were taken during the spring season of 2014 (September 2014). From all sites, water samples were collected in sterile 1 L Schott glass bottles (DWK Life Sciences, Mainz, Germany) and sediment samples (except for Lekkerrus) in sterile plastic 50 mL Falcon tubes (Life Technologies, Johannesburg, South Africa) and processed separately. Sediment samples were collected 0.5 m below the water line. All samples were taken directly from the hot spring water without any human contact, except for sampling at Lekkerrus where the water samples were collected from a pipe that ran directly into the swimming pool. The flow within the pipe was manually controlled depending on demand, that ranged from once a day to once a week. Samples were transported to the laboratory in a cooler box kept at 4 °C, and processed within 72 h of collection.

### 2.2. Isolation of Bacteria

For isolation of bacteria from the water samples, a 100-mL sample aliquot was passed through a 0.22 µm membrane filter (Millipore nitrocellulose GSWP 04700, Merck, Modderfontein, South Africa) and the membrane filters were then placed on the surface of five different agar media prepared as per manufacturer’s instructions: nutrient agar, actinomycete isolation agar, minimal Luria agar, cyanobacterial agar and potato dextrose agar (HiMedia, Mumbai, India). Minimal Luria agar was made up as 10% of the recommended concentration normally used to create a low nutrient media simulating the low nutrients in the water samples. A total of four plates per media were inoculated. The filters were removed before the plates were incubated aerobically for 48 h at 37 °C and 55 °C. For isolation of bacteria from sediment samples, the streak plate method was used; agar plates were streaked using an inoculation loop to spread out the samples, and single colonies were picked for pure cultures. All media were incubated without inoculation concurrently as a control for media sterility and contamination. All colonies with distinct and different morphology were sub-cultured at least three times until pure cultures were obtained and cultures were maintained on nutrient agar slants at 4 °C. Since this was a study on microbial diversity and not a quantitative investigation, colonies were picked based on differences in colony morphology. The isolation conditions for 16 isolates are described in Table 2.

### 2.3. DNA Extraction, 16S rDNA Gene Sequencing and Phylogeny

The DNA was extracted by re-suspending bacterial colonies in sterile phosphate buffer saline, then boiled for 10 min and centrifuged at 10,000 rpm for 10 min in a C2500 Prism Air-cooled Microcentrifuge (Labnet International, Edison, NJ, USA) [46]. The supernatant was used for polymerase chain reaction (PCR). Universal primers 8F, 27F, 300F and 1472R [48] for the complete 1400 bp 16S ribosomal DNA (rDNA) gene fragment were obtained from Inqaba Biotechnology (Pretoria, South Africa) and used in different combinations to obtain a PCR product for direct sequencing. The PCR tube per sample contained 9.5 µL of water, 12.5 µL of PCR Master Mix (2×) (DreamTaq Green K1081; Thermo Fisher Scientific, Johannesburg, South Africa), 1 µL of each primer at 10 µM, and 1 µL of genomic DNA at 1–10 ng. A “water only/no DNA” control was included in each run, as a no-template control (NTC), to ensure contamination had not occurred. The PCR assays were run on a Bio-Rad MyCycler (Bio-Rad, Rosebank, South Africa). The thermal cycle profile was as follows: initial denaturation at 94 °C for 5 min, 40 cycles of 94 °C for 30 s, 50 °C for 30 s and 72 °C for 60 s, followed by 72 °C for 10 min for the final extension, and held at 4 °C until the machine was switched off. The PCR products were run on 1% tris-acetate-ethylenediaminetetraacetic acid (TAE) agarose gels at 80–100 V for 60 min together with molecular weight markers (SM1113 middle markers; Thermo Scientific Waltham, MA, USA), stained with ethidium bromide and visualised and photographed with the Bio-Rad Gel Doc EZ Imager (Bio-Rad). The PCR products were then sequenced with the ABI BigDye Terminator v3.1 cycle sequencing kit (Applied Biosystems, Foster, CA, USA) according to the manufacturer’s instructions, and run on the ABI capillary sequencer at the African Centre for DNA Barcoding, University of Johannesburg. If primer 8F failed, only partial sequencing was obtained. A contiguous sequence was constructed with forward and reverse sequencing data resulting in a fragment of approximately 1400 bp, with DNA Baser Sequence Assembler v4 (2013) (Heracle BioSoft, Arges, Romania).

Sequences were compared with those in the NCBI database (GenBank) using the BLAST Sequence Analysis Tool [49], and EzTaxon-e [50]. Isolates with a >99% match to the published sequences were identified to the species level, and those with a >97% match were identified to the genus level [51]. The highest similarities (as percentage similarities) and accession numbers are given in Table S2 (Supplementary Materials). Sequences of type strains were obtained from GenBank and included in phylogenetic analysis to confirm identification. Alignments were made by Clustal Omega, a multiple sequence alignment programme (European Bioinformatic Institute, Cambridge, UK). Neighbour-joining phylogenetic trees of a 947 bp fragment for Actinobacteria and a 510 bp fragment for Proteobacteria were constructed using the SeaView software program [52]. Methanogenic bacteria (GenBank DQ372975.1), were found to be the outgroup in the Proteobacteria tree, while the Actinobacteria tree was unrooted for better resolution. Statistical confidence in branching points was determined by bootstrap analysis. Complete and partial sequences were submitted to GenBank, and the accession numbers are indicated in Table S2 (Supplementary Materials). GenBank accession numbers of the type strains used in the phylogenetic trees are listed in Table S3 (Supplementary Materials).

The consensus sequences were compared to those listed in two databases, GenBank and EzTaxon-e. The highest similarities (as percentage similarities) and accession numbers are given in Table S2 (Supplementary Materials). Values >95% suggest a match in genus, while a value of >99% suggests a match in species. Similarities of 86–95% can only be identified to a family level [51]. Partial sequences of the 16S rDNA sequences were used for Actinobacteria (947 bp) and Proteobacteria (510 bp) phylogeny, allowing alignment to the shortest fragment of the group. Actinobacteria sequences of a 947 bp fragment of the 16S rDNA were analysed as an unrooted parsimony tree (SeaView) with bootstrapping, after alignment using Multiple Sequence Comparison by Log-Expectation (MUSCLE).

### 2.4. Assessment of the Presence of Legionella spp. Using Real-Time PCR

A 300-mL sample aliquot of hot spring water was passed through a 0.22 µm filter, washed off with phosphate buffered saline, centrifuged at 10,000 rpm in a C2500 Prism Air-cooled Microcentrifuge, and the DNA extracted with ZR fungal/bacterial DNA MiniPrep kit (D6005) (Zymo Research Corp., Irvine, CA, USA). The presence of *Legionella* spp. in the water samples was investigated using real-time PCR on a Corbett Life Science Rotor-Gene 6000 Cycler (Qiagen, Hilden, Germany). Primers used in the PCR assay were JFP (5′-AGG GTT GATAGG TTA AGA GC-3′) and JRP (5′-CCA ACA GCT AGT TGACAT CG-3′) [53]. The PCR assay was run in a total volume of 20 µL containing 10 µL of 2× SensiFAST HRM mix (Bioline, Luckenwalde, Germany), 0.2 µL of each primer (each at a final concentration of 0.2 µM), 5.6 µL of nuclease-free water and 4 µL of template DNA. Template DNA from *L. pneumophila* ATCC 211-33-2 (American Type Culture Collection (ATCC), Manassas, VA, USA) was used as positive control. To test the detection limit of the PCR reaction, the DNA concentration of the positive control was determined using a NanoDrop Hach 6200 Spectrophotometer (Hach, Loveland, CO, USA), and then serially diluted up to 10^−6^. The samples and the positive control were run in triplicate.

Reaction conditions for the PCR were optimised as follows: initial activation at 95 °C for 10 min; denaturation at 95 °C for 5 s; annealing at 57 °C for 5 s; and a final extension at 72 °C for 5 s, for a total of 40 cycles. The last cycle was followed by a second incubation period of 5 min at 72 °C. The second incubation was followed by a melt curve prepared by ramping up the melting temperature from 72 °C to 95 °C at a ramp rate of 1 °C at each step, a pre-melt hold of 90 s on the 1st step followed by a 5-s hold on each of the next steps. A Rotor-Gene 6000 Cycler (Qiagen) was used to run the PCR assays. Reaction mixtures without template DNA were used as NTCs in each reaction. Melting-curve analyses were performed using the Rotor-Gene Real-Time Analysis Software, Version 6.1 (Build 93) (Corbett Life Science, Sydney, Australia).

### 2.5. Antibiotic Resistance Assay

The following 10 antibiotics (obtained from Mast Diagnostics, Merseyside, UK, if not indicated otherwise) were used in this study, representing six different classes: β-lactam (carbenicillin, CAR), aminoglycosides (gentamycin, GEN; kanamycin, KAN; streptomycin, STR), tetracycline (TET), amphenicols (chloramphenicol, CHL; ceftriaxone, CEF), sulphonamides (co-trimoxazole, COT) and quinolones (nalidixic acid, NA; norfloxacin, NOR), and assayed using the Kirby-Bauer disk diffusion assay [54] according to the criteria published by the Clinical Laboratory Standards Institute [55]. Commercial antibiotic discs from Oxoid (Thermo Fisher Scientific) were used at the following potencies in µg: gentamycin 10, tetracycline 30, co-trimoxazole 25, chloramphenicol 30, ceftriaxone 30 and norfloxacin 10. Solutions of carbenicillin (100 µg), kanamycin (30 µg), streptomycin (10 µg) and nalidixic acid (125 µg) were prepared from stock solutions (Sigma-Aldrich, Johannesburg, South Africa) and dried on sterile Whatman No 17 discs (Thermo Fisher Scientific) before placement on a lawn of bacteria. The inoculum (100 µL) was prepared from an overnight culture in nutrient broth. The discs were applied to lawns of bacteria on Mueller-Hinton agar (HiMedia, http://doua.prabi.fr/software/seaview). Plates were scored after 24 h at 53 °C or 37 °C, depending on the isolate. The zones of inhibition were measured and compared to the CLSI standard values. The multiple antibiotic resistance (MAR) index was calculated as the ratio (a:b) between the number of antibiotics to which the isolate was resistant (a) and the total number of antibiotics tested (b) [56]. MAR was defined as the resistance of isolates to at least three different antibiotics.

### 2.6. GenBank Accession Numbers

The 16S rDNA gene sequences of hot spring isolates from South Africa were allocated accession numbers and deposited in Genbank as indicated in Table S2 (Supplementary Materials). Strains of *Actinobacteria*, *Kocuria* sp. and *Arthrobacter* sp. have accession numbers MF120234 and MF120235, respectively. Two alpha Proteobacteria were assigned numbers MF120236 and MF120239. Accession numbers: MF120227–MF120233 and MF120237 were given to beta *Proteobacteria* including *Gulbenkiania mobilis*, *Cupriavidus gilardii*, *Tepidimonas fonticaldi* and *Ralstonia mannitolilytica*. Four strains of gamma Proteobacteria including *Hafnia alvei* have Genbank accession numbers of MF144571–MF144573 and MF120238. All reference strains used for analyses are listed in Table S3 (Supplementary Materials) together with their associated Genbank accession numbers.

## 3. Results

### 3.1. Isolation Rates of Bacteria

**Figure 1:**
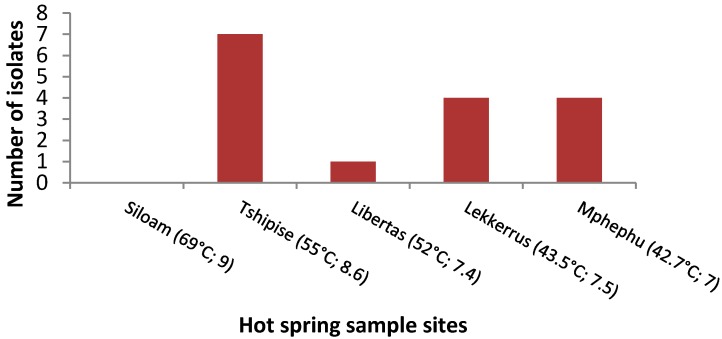
Distribution of the number of isolates from five hot springs, Limpopo Province, South Africa. Temperature and pH of water samples are given in parentheses.

The majority of bacteria were isolated from water samples, although two isolates, namely 42T and 44M, were from the sediment. Bacteria were not isolated from the warmest hot spring, Siloam, at 69 °C, while the second warmest site, Tshipise, at 55 °C, had the highest number of isolates (Figure 1).

**Figure 2:**
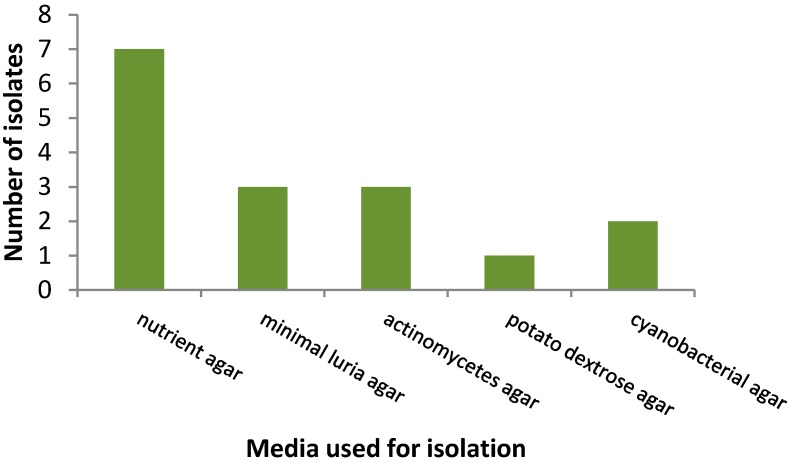
Comparison of number of isolates obtained on different media.

Furthermore, the coldest water temperature at Mphephu at 42.7 °C did not yield the highest number of isolates. By comparing the different media used for isolation, nutrient agar performed the best, resulting in the highest number of isolates, and potato dextrose agar, which is generally used for the isolation of fungi, was the poorest performer (Figure 2).

However, this is only an observation limited to this specific sampling regimen. Other isolation patterns on media might be observed in different sampling programmes. Furthermore, as *Bacillus* spp. and related species were isolated from the same plates, isolates could have competed for space and nutrients.

### 3.2. 16S rDNA Gene Sequencing

As indicated in the Supplementary Materials (Tables S1–S3), isolates of *Sphingomonas echinoides*, *Hafnia alvei*, *Tepidimonas fonticaldi*, *Gulbenkiania mobilis*, *Ralstonia mannitolilytica*, *Cupriavidus gilardii*, *Kocuria turfanensis* and *Arthrobacter luteolus* were identified to the species level, while isolates of *Cronobacter*, *Silanimonas*, *Tepidimonas* and *Zoogloea* were identified only to the genus level. Four isolates (55M, 59Lk, 61T and 72T) were unknown as they presented poor matches to the published sequences. Sequences were submitted to GenBank (List S1; Supplementary Materials), and their relevant accession numbers are listed in the Supplementary Materials.

### 3.3. Phylogenetic Analysis

**Figure 3:**
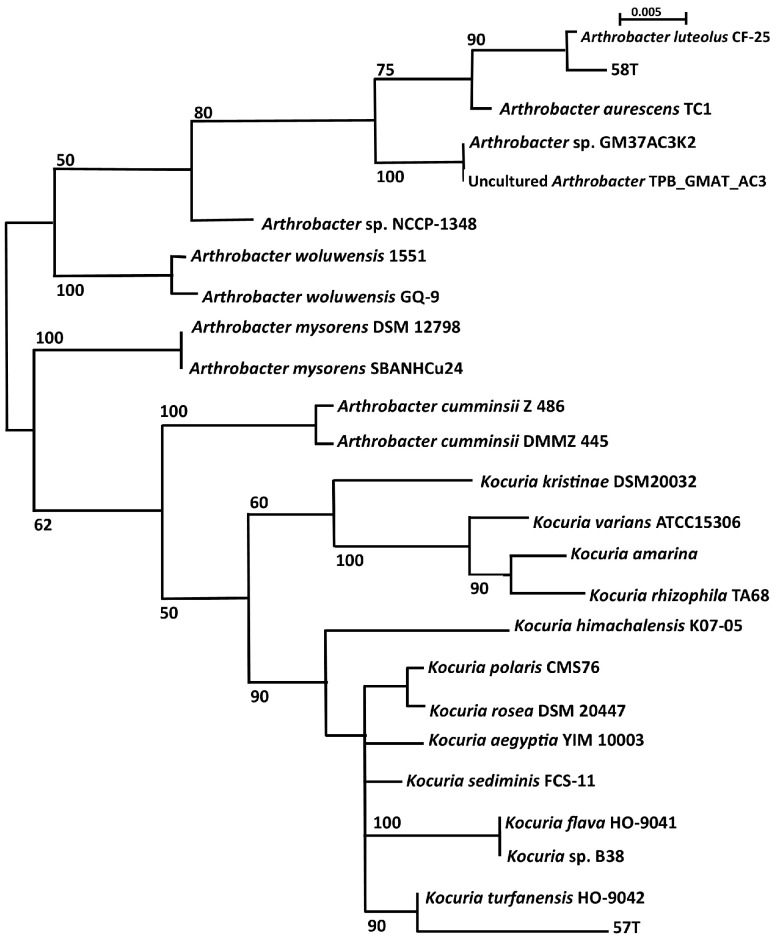
Unrooted parsimony tree for Actinobacteria showing the placement of isolate 57T with *Kocuria* and isolate 58T with *Arthrobacter* with bootstrap values.

Two isolates identified in this investigation, namely 57T (pink pigment) and 58T (yellow pigment), grouped with *K. turfanensis* and *A. luteolus*, respectively (Figure 3), as predicted from the BLAST results with GenBank and EzTaxon-e, given in the Table S2 (Supplementary Materials). Reference strains are included in the analysis (accession numbers listed in Table S3; Supplementary Materials), and pathogenic isolates are *Arthrobacter woluwensis*, *Arthrobacter cumminsii*, *Arthrobacter mysorens*, *Kocuria rhizophila*, *K. marina*, *K. varians*, *K. kristinae* and *K. rosea* [18]. Three unknown sequences from hot-spring isolates identified in other studies, and listed in the GenBank database, were included. *Arthrobacter* NCCP from hot springs in Pakistan grouped with the pathogenic group of *A. woluwensis*. Uncultured *Arthrobacter* clone BR5clone TPB_GMAT_5_1 grouped with the cultured *Arthrobacter* GM37 isolate from hot springs in India, suggesting that they could be closely related. *Kocuria* B38, an isolate from hot springs in India, grouped with *K. flava*.

Following MUSCLE alignment (SeaView), a parsimony tree of the 16S rDNA gene sequences of the Proteobacteria isolates was drawn using a partial sequence of 510 bp, with bootstrapping. A methanogenic archaeon (GenBank accession No. DQ372975.1) was the outgroup used. The results of this investigation showed that three classes of the Proteobacteria were represented, namely Alphaproteobacteria (*n* = 2), Betaproteobacteria (*n* = 8), and Gammaproteobacteria (*n* = 3). Reference type strains were included in the analysis (Table S3; Supplementary Materials) and the opportunistic pathogens are *Sphingomonas paucimobilis*, *Cronobacter sakazakii*, *Hafnia alvei* and *H. paralvei*, *Tepidimonas arfidensis*, *Ralstonia mannitolilytica* and *Ralstonia pickettii*, *Cupriavidus metallidurans* and *C. gilardii*.

From published sequences in GenBank, four isolates that have not been cultured but sequenced from hot springs in other studies were included in the phylogenetic tree to establish whether they were similar to isolates obtained in this study, or to determine whether their identity could be further characterised.

### 3.4. Detection of Legionella spp.

A total of four water samples were analysed in triplicate. All samples analysed were negative for the 16S rDNA gene amplified. A real-time PCR assay for *Legionella* is given in Figure S2A (Supplementary Materials) while Figure S2B (Supplementary Materials) shows the high-resolution melt (HRM) curve analysis of the PCR reaction. The mean melting temperature of the positive samples was 86.1 ± 0.1 °C.

### 3.5. Antibiotic Resistance

Isolates *Kocuria*, *Arthrobacter* and *Hafnia* spp. were resistant to two antibiotics in different combinations of ceftriaxone, nalidixic acid and carbenicillin, as indicated in Table 3. Isolate *Cronobacter* spp. was sensitive to all ten antibiotics while the unknown isolate (isolate 72T) belonging to the family Enterobacteriaceae, was also resistant to two antibiotics, namely chloramphenicol and ceftriaxone.

## 4. Discussion

Phylum Proteobacteria (mostly beta and gamma Proteobacteria) dominates the microbiome of Tshipise and Mphephu (up to 30%), while the phylum Actinobacteria was found to occur at less than 1% [54]. However, metagenomic studies report diversity as proportions of different DNA sequences without any information on the viability of cells. In fact, Gram-positive spore-forming *Bacillus* of the phylum Firmicutes are the most predominant bacteria isolated from hot springs globally [32,33,35], although there are sporadic reports of isolation by culture of *Pseudomonas* spp. of the phylum Proteobacteria in Algeria [57] and Iceland [58].

### 4.1. Phylum Actinobacteria

From sediment samples, two pigmented mesophilic isolates, namely 57T and 58T, were identified to be *Kocuria turfanensis* and *Arthrobacter luteus*, respectively, using the search tool, BLAST for GenBank and EzTaxon-e (Table S2; Supplementary Materials) and confirmed using phylogenetic analysis (Figure 3).

*Kocuria* spp. ASB107, closely related to *K. polaris* and *K. rosea*, which was isolated from Iranian Ab-e-Siah hot springs, showed resistance to gamma and ultra violet (UV) rays with a temperature growth range of 0 to 37 °C [39]. Another isolate of *K. rosea* MG2 from the same Iranian hot spring optimally grew at pH 9. 2 and at a temperature of 28 °C. It could also tolerate 15% NaCl salinity, high doses of radioactivity and grew in the presence of 4% hydrogen peroxide [40], providing further proof that members of this genus are capable of multiple-extreme resistance. An unpublished *Kocuria* spp. B38 was isolated from Bakreshwar hot springs in India and was included in the phylogeny tree to establish whether there was any similarity between that one and the one isolated during this investigation. *Kocuria* B38 grouped with *Kocuria flava*, an airborne *Actinobacteria* [59] GenBank KC492107, with a bootstrap value of 90%, suggesting that it is possible to culture closely related *Kocuria* isolates that were previously reported uncultured from hot springs at other sites. Pathogenic *Kocuria* sp. are *K. rhizophila*, *K. marina*, *K. rosea* listed in reviews [18,60], and *K. kristinae* [61,62] and *K. varians* [63]. All, except for *K. rosea*, fell into a separate group. Even though neither *K. turfanensis* nor *K. flava* has been reported as opportunistic pathogens, it does not exclude the possibility that they also have the ability to acquire virulence factors.

Similarly, radio-resistant *Arthrobacter* sp. has been isolated from hot springs in Japan [64] and Tibet [65]. Three 16S rDNA gene sequences from *Arthrobacter* sp. isolated from hot springs were included in the phylogenetic analysis (Figure 3). Interestingly, *Arthrobacter* NCCP 1348 from Pakistan (GenBank Accession LC065375) was related to pathogenic *A. woluwensis*, while *Arthrobacter* sp. GM37AC3 K2 strongly grouped with uncultured *Arthrobacter* sp. clone TPB_GMAT_AC3. This latter observation was not surprising since both came from the same investigation of hot springs in India (Table S3; Supplementary Materials). Three species of pathogenic *Arthrobacter* were included in the phylogenetic analysis and *A. woluwensis*, and *A. mysorens* were closely related. Interestingly, *A. cumminsii* grouped closed with the pathogenic *Kocuria* species. A literature search did not reveal *A. luteolus* as an opportunistic and emerging pathogen; however, the type strain of *A. luteolus* was isolated originally from human clinical specimens [66], and therefore this does not entirely exclude this species from being a potential opportunistic pathogen.

### 4.2. Phylum Proteobacteria

Fourteen Proteobacteria were represented by three classes: Alphaproteobacteria (*n* = 2), Betaproteobacteria (*n* = 8) and Gammaproteobacteria (*n* = 4) (Table S2; Supplementary Materials). Of these, four isolates could not be classified up to genus level as they were <97% similar to GenBank database entries. Four could be identified to the genus level, and six identified to the species level.

#### 4.2.1. Alphaproteobacteria

Isolate 87T was similar to *Sphingomonas echinoides* >99% and could, therefore, be identified to species level. However, isolate 61T had a poor BLAST match (92%) and remains an unknown member of the Alphaproteobacteria. *Sphingomonas* sp. has been isolated in the environment of soil [67], pristine rivers [62] and from hot springs in China [68]. *Sphingomonas echinoides* is related to but different from the pathogen *S. paucimobilis* (Figure 3).

#### 4.2.2. Betaproteobacteria

Within this class, *G. mobilis* (isolate 27M), *R. mannitolilytica* (isolate 69Lk) and *C. gilardii* (isolate 31Lk) were identified down to species level. Isolates 37LB and 5T could only be identified to a genus level as *Tepidimonas* sp. and *Zoogloea* sp. respectively. Two isolates, namely 55M and 59Lk, remain unknown due to their low match (<97%) with published data as indicated in (Table S2; Supplementary Materials). Opportunistic pathogens within this class of Betaproteobacteria are included in the phylogeny tree (Figure 3) as *R. mannitolilytica* [18] and *R. pickettii* [69], *C. metallidurans* [70] and *C. gilardii* [71] and *T. arfidensis* [72]. *Gulbenkiania mobilis* has not been reported to be an opportunistic pathogen. As a result, three of the four isolates identified to species level have been reported as opportunistic pathogens. As indicated in Table S4 (Supplementary Materials), these genera have been previously isolated from hot-spring environments, suggesting that these common environmental bacteria have the capacity to adapt to harsh conditions of high temperature. *Tepidimonas* has been reported as thermophilic, growing optimally at 55 °C [73] and *Sphingomonas* as chlorine resistant [74]. *Cupriavidus* has been isolated from drinking water [22], but not from hot springs.

The presence of unknown bacteria further supports the need to study such environments to contribute to the knowledge bacteria diversity in hot-spring niches. Including the two unknown isolates from this investigation, sequences of four Betaproteobacteria isolates from hot springs (*G. mobilis* V28, *Cupriavidus* NCCP, uncultured *Tepidimonas* clone TP and *Tepidimonas* BR5) were obtained from GenBank and included in the phylogenetic analysis to establish whether there was a similarity with isolates in this study or other reference strains. *Gulbenkiania mobilis* V28 from Indian hot springs was similar to our *G. mobilis* isolate 27M. No further identification to a species level could be obtained with *Cupriavidus* NCCP and uncultured *Tepidimonas* sp. clone TPB_GMAT_5_1. Isolate 42T which was identified as *T. fonticaldi*, grouped closely with *Tepidimonas* BR5, an isolate from hot springs in India. By extending the study to include retrospective 16S rDNA genetic data from other investigations of hot springs, a broader knowledge of the diversity of culturable hot-spring microorganisms can be gained.

#### 4.2.3. Gammaproteobacteria

This class includes *Cronobacter*, *Hafnia*, *Silanimonas* and *Legionella*. Isolate 79M was identified as *H. alvei*, isolate 80Lk as *Cronobacter* sp. and isolate 44M as *Silanimonas* sp. Isolate 72T was the single unknown in this group, with a match of only 89% identity with the *Cronobacter* sp. sequence in the public domain. Gammaproteobacteria, especially *Legionella*, have been isolated from hot springs. Other Gammaproteobacteria isolated from hot springs worldwide are listed in Table S4 (Supplementary Materials), including pathogenic *C. sakazakii* in Malaysian hot springs [75]. The reference strains of Gammaproteobacteria opportunistic pathogens in the phylogenetic tree are indicated as *H. alvei*, *H. paralvei* and *C. sakazakii* (Table S3; Supplementary Materials). *Hafnia paralvei* formerly called *H. alvei* [76] is the only species in this genus.

It is paradoxical that *L. pneumophila* is highly sensitive but still able to persist in the environment [38]. Investigators have also observed that there is no correlation between *Legionella* loads in water and temperature [77,78]. Although the aim of this study was to investigate the diversity of culturable microbes from hot springs, the investigation of possible *L. pneumophila* contamination using RT-PCR was included because *Legionella* is the most common opportunistic pathogen of hot-spring waters [11]. The sensitivity of the assay was 0.006 ng/µL which excludes the possibility of lack of detection because of the assay method used. There are several possible reasons to explain the lack of detection of *Legionella* in this study. From the literature, not all hot springs have been reported positive for *Legionella*, being as low as 3% in Japan [79] to as high as 51% in China and 71% in Thailand. It is possible that the sample size was too small in this study, or that the presence of *Legionella* could be seasonal. Seasonal variations have been reported in wastewater sites in French river watershed samples [29] as well as water reservoirs in Taiwan [80]. A follow-up investigation could include biofilm and sediment samples as *Legionella* replicates in protozoa that naturally colonize and persist in biofilms [81].

### 4.3. Antibiotic Resistance of Opportunistic Emerging Pathogens

Antibiotic resistance profiles were determined for five isolates (57T, 58T, 72T, 79M and 80Lk). Of those, four exhibited antibiotic resistance to two antibiotics, with one isolate being completely sensitive to the ten antibiotics tested. All four isolates differed regarding the antibiotics they were resistant to, and combinations of resistance towards, CEF, NA, CAR and CHL was observed. Resistance to CEF was reported in three of the four isolates while only one isolate showed resistance to CHL.

Antibiotic resistance of emerging opportunistic bacteria is poorly investigated [60]. However, the results from this study corroborate previous findings to a certain degree. *Kocuria* sp. (isolate 57T) was resistant to CEF and NA and sensitive to CHL, TET, STR, GEN and CAR in this study. In a review of antibiotic resistance in *Kocuria* [60], these same results were also found by other researchers. However, variability resistance was also reported to COT and KAN in previous studies, suggesting that *Kocuria* spp. are able to acquire additional resistance genes than that reported in this study. Similarly, *Hafnia* spp. was reported to be resistant to CEF and sensitive to GEN, as found in this study with isolate 79M, however TET and CHL resistance have been previously reported [82] again implying that *Hafnia* spp. are also able to obtain further antibiotic resistant mechanisms. The lack of resistance to any antibiotic of *Cronobacter* sp. isolate 80Lk is consistent with that found in other studies of *Cronobacter* from foods in Brazil [83]. In a larger study of *Cronobacter sakazakii* from 78 domestic kitchens in the US, 67% were TET resistant and 48% NA resistant. All were sensitive to GEN. [84]. This suggests that multi drug resistance is highly likely in this group of bacteria. Antibiotic resistance of emerging opportunistic pathogens is important for two reasons. Firstly, it has implications in that should these isolates cause infections, therapy will be limited to the antibiotics to which they are sensitive. Secondly, by monitoring the antibiotic resistance of environmental opportunistic microbial populations, one can also track these microbes as the etiological agent of an infection based on their antibiotic resistance profiles, to determine the source of nosocomial or community outbreaks.

This study provides evidence of the presence of different emerging opportunistic pathogens in hot springs in Limpopo Province, South Africa. To establish whether these bacteria have a significant impact on human health, the literature on nosocomial infections, opportunistic infections of HIV patients and cystic fibrosis patients was reviewed. With the exclusion of *Legionella*, the reviews on opportunistic infections in HIV patients [85,86,87] and opportunistic nosocomial infections [24,25] do not mention the abovementioned bacteria identified in this study. However, *Ralstonia* sp. was mentioned as a risk factor for cystic fibrosis patients [69,88].

### 4.4. Relationship between Waterborne Pathogens and Emerging Opportunistic Pathogens

The question must be asked that if these opportunistic bacteria listed in (Table S1; Supplementary Materials) do cause infections, then why are these not reflected in the literature reviews of waterborne pathogens? In fact, these environmental bacteria have been described in the literature as “rarely isolated, strange, atypical, uncommon and unusual” [9,89,90,91,92] pathogens, which could provide a clue of why these bacteria are seemingly overlooked or underestimated in the diagnosis as aetiological agents for infections. A more thorough literature search could confirm the significance of these microorganisms when recognised as being pathogenic; however, the sporadic reports listed in Table S1 (Supplementary Materials) should be of some concern and alarm for clinicians, healthcare workers and medical diagnostic laboratories. It is possible that these bacteria are simply not monitored because they are regarded as “harmless” in a healthy individual. However, this benchmark should be flexible to cover pathogens that may be emerging for a particular population, such as a population with high levels of malnutrition, a high incidence of HIV/AIDS, and a large number of very young or elderly age groups. The criteria for designation of a pathogen as an emerging pathogen are often vague. Pathogens are classified according to their level of biological hazard (biohazard) [22]. The United States’ Centers for Disease Control and Prevention (CDC) categorises various diseases in levels of biohazard, Level 1 being minimum risk and Level 4 being extreme risk. Species classified as Biohazard Level 2 cause mild diseases which are unlikely to spread in the human population and for which an adequate therapy exists; they can be used safely for routine diagnostic works. *Legionella*, *C. sakazakii*, *H. alvei*, *A. luteolus*, *Cupriavidus* and *R. mannitolilytica*, fall into this category. Biohazard Level 1 microbial species are non-infectious, and precautions are minimal. The Level 1 species have been demonstrated by in vitro and in vivo testing to be non-pathogenic. *Tepidimonas*, *Kocuria*, *Gulbenkiania* and *Zoogloea* fall into this lowest biohazard classification; however, they are also able to cause opportunistic infections. For example, Lewis [93] studied urinary tract infections in South Africa, and *Kocuria* sp. was isolated and cultured but was disregarded as the etiological agent, rightly so according to Van Belkum’s classification [22]. However, since this is in flux, the classification should constantly be reviewed and updated. Reviews of emerging infectious diseases published after the Netherlands Commission on Genetic Modification (COGEM) research report had appeared in 2011 [22], named *Sphingomonas* [94], *Ralstonia* [95] and *Kocuria* [18,19]. Another reason why these bacteria may have rarely been mentioned as pathogens is misidentification as *Pseudomonas*. *Sphingomonas echinoides* was initially called *Pseudomonas echinoides* [96] while *Ralstonia* was also previously called *Pseudomonas* [97].

Metagenomic studies of these hot springs in Limpopo [98], indicated that 0–20% of the sequences were “unclassified bacteria” suggesting that there is a great diversity of bacteria that has yet to be discovered and characterised. Four unknown bacteria were sequenced in this study (61T, 55M, 59Lk and 72T) as indicated in Table 3 (Supplementary Materials), and eight sequences (four Actinobacteria and four Proteobacteria) of partially identified isolates from other investigations of hot springs, were obtained from GenBank allowing comparison of 16S rDNA gene data. With such analysis, the uncultured *Arthrobacter* sp. clone TPB_GMAT_AC3 was found to be like *Arthrobacter* sp. GM37AC3 K2, and *Arthrobacter* sp. NCCP-1348, isolated from a hot spring in Pakistan, was closely related to pathogenic *A. woluwensis* (Figure 3). *Kocuria* B38, from a hot spring in India, grouped with *K. flava*, an airborne bacterium from China, with a bootstrap value of 100%, suggesting that it was *K. flava.* This shows the value of 16S rDNA gene sequence data in identification of unknown bacteria in retrospect. Such information could give a broader view of the presence of potential opportunistic pathogens in other hot springs, and an understanding of how they may enter the water system via airborne or soil routes. Improvements could be made to this investigation by using different isolation culture media or enrichment methodology, and laboratory conditions to extend the number of isolates obtained. The dissolved oxygen content of hot springs is negligible, and therefore we expect to find a large proportion of anaerobic microbes.

## 5. Conclusions

This study is the first report about cultured isolates from hot springs in the Limpopo Province. The study confirmed the presence of the phyla Actinobacteria and Proteobacteria. The phylum Actinobacteria was represented by two genera (*Kocuria* and *Arthrobacter*). The phylum Proteobacteria was represented by three classes: Alphaproteobacteria (*Sphingomonas*), Betaproteobacteria (*Zoogloea*, *Gulbenkiania*, *Cupriavidus* and *Tepidimonas*) and Gammaproteobacteria (*Ralstonia*, *Silanimonas*, *Hafnia* and *Cronobacter*). Although *Legionella* is considered a re-emerging pathogen, it was not detected in the waters of these hot springs.

The organisms isolated in this study are commonly found environmental bacteria and have been reported to possess the capacity to cause infections in humans, especially in people with a weakened immune system. Thus, this study highlights the potential of these hot springs to serve as reservoirs for emerging opportunistic pathogens. Given the wide use of spring waters by humans for drinking and domestic purposes, the results of this study are relevant for public health officials to understand the possible aetiology of less reported infections caused by emerging opportunistic pathogens as these could cause disease outbreaks where a high proportion of individuals are immuno-vulnerable. Such information would be valuable to put in place mechanisms to protect populations from infections related to pathogens in hot springs.

## Figures and Tables

**Table 1:** Geographical location of sampling sites and sampling conditions.

Sampling Site	GPS Location	pH	Temperature (°C)	Comments
Tshipise	22°36.521′ S 30°10.345′ E	8.63	55.2	Open to air in enclosed section
Siloam	22°53.667′ S 30° 11.7718′ E	9	69	Exiting from pipe on private property
Mphephu	22°54.225′ S 30° 10.83′ E	7.07	42.4	Open to air in enclosed section
Lekkerrus	24°28.04′ S 28°33.1′ E	7.46	43.5	Pipeline conveys water into pool; flow manually controlled
Libertas	24°27′36″ S 28°34′11″ E	7.44	52.1	Water pumped at source

GPS: global positioning system.

**Table 2:** List of Actinobacteria and Proteobacteria isolates with isolation conditions given.

Isolate No.	Site	Isolation Temperature (°C)	Sample	Isolation Media
57T	Tshipise	37	Water	Nutrient agar
58T	Tshipise	37	Water	Actinomycete isolation agar
87T	Tshipise	25	Water	Minimal Luria agar
61T	Tshipise	25	Water	Cyanobacterial agar
72T	Tshipise	37	Water	Nutrient agar
80Lk	Lekkerrus	37	Water	Nutrient agar
79M	Mphephu	37	Water	Nutrient agar
44M	Mphephu	53	Sediment	Nutrient agar
55M	Mphephu	37	Water	Potato dextrose agar
37Lb	Libertas	53	Water	Actinomycete isolation agar
42T	Tshipise	53	Sediment	Nutrient agar
59Lk	Lekkerrus	37	Water	Minimal Luria agar
5T	Tshipise	53	Water	Minimal Luria agar
27M	Mphephu	53	Water	Actinomycete isolation agar
69Lk	Lekkerrus	25	Water	Cyanobacterial agar
31Lk	Lekkerrus	53	Water	Nutrient agar

**Table 3:** Antibiotic resistance profiles of Actinobacteria and Proteobacteria isolates against ten different antibiotics.

Isolate No.	Identification	CAR100	GEN10	KAN30	STR10	TET30	CEF30	CHL30	COT25	NA30	NOR10
57T	*Kocuria turfanensis*	15	9	12	7	12	0	14	16	0	2
58T	*Arthrobacter luteolus*	0	5	3	4	9	1	6	11	0	2
79M	*Hafnia alvei*	0	2	3	1	4	0	5	6	3	7
80Lk	*Cronobacter* sp.	1	4	5	5	6	2	6	6	3	11
72T	unknown Enterobacteriaceae	5	6	10	5	7	0	0	16	5	14

CAR: Carbenicillin; GEN: gentamicin; KAN: kanamycin; STR: streptomycin; TET: tetracycline; CHL: chloramphenicol; CEF: ceftriaxone; COT: co-trimoxazole; NA: nalidixic acid; NOR: norfloxacin; all in µg. Values of 0 indicate resistance while numerical values are zones of inhibition in millimetres.

## Supplementary Materials

The following are available online at www.mdpi.com/1660-4601/14/9/1070/s1, Figure S1: Bacteria isolated from various hot springs worldwide, Table S1: Waterborne opportunistic bacterial pathogens, Table S2: Closest match of 16S rDNA gene sequence from hot-spring isolates with GenBank and EzTaxon-e with percentage similarity and associated accession numbers, Table S3: List of the accession numbers of all the strains from GenBank used in phylogenetic analysis, Table S4: List of emerging opportunistic pathogens that have been isolated from hot-spring water, List S1: Raw sequence data to be submitted to GenBank.
