# Supplementary material for: Phylogenetic Analysis and Antimicrobial Profiles of Cultured Emerging Opportunistic Pathogens (Phyla Actinobacteria and Proteobacteria) Identified in Hot Springs
Source: Int J Environ Res Public Health. 2017 Sep 15;14(9):1070. doi: 10.3390/ijerph14091070 (PMC5615607; doi:10.3390/ijerph14091070)
Supplement: Supplementary file 1 [file ijerph-14-01070-s001.pdf]

Supplementary Materials for Phylogenetic Analysis and Antimicrobial Profiles of Cultured Emerging Opportunistic Pathogens (Phyla Actinobacteria and Proteobacteria) Identified in Hot Springs

Jocelyn L. Jardine, Akebe Luther King Abia, Vuyo Mavumengwana and Eunice Ubomba-Jaswa

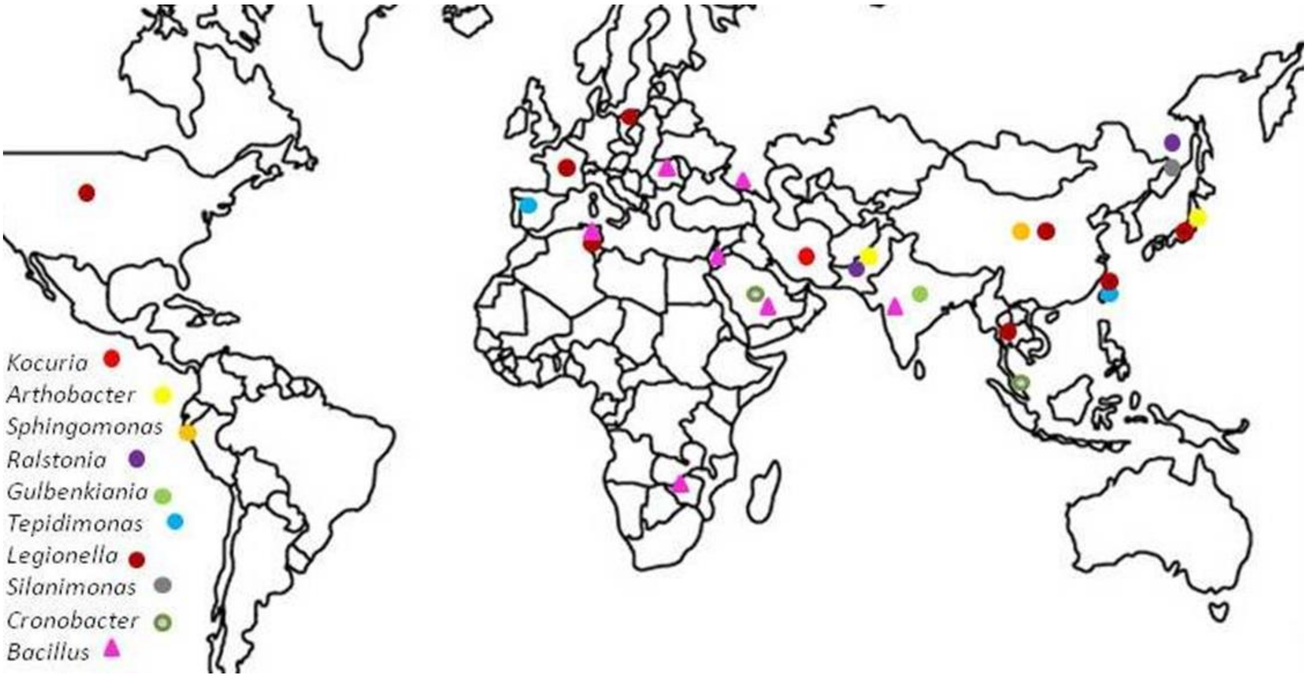

Figure S1. Bacteria isolated from various hot springs worldwide

Table S1: Waterborne emerging opportunistic bacterial pathogens

PATHOGEN	INFECTION	HOST	LOCATION	REFERENCE
Sphingomonas sp.	pneumonia	epidemic in intensive care unit patients	Turkey	[96]
Sphingomonas	diabetes, alcoholism, nosocomial and catheter-related bacteraemia	community acquired and nosocomial	Taiwan	[92]
Sphingomonas paucimobilis	bacteraemia in hypertension diabetic male with kidney and heart disease	immunocompromised	India	[21]
Ralstonia pickettii and *R. mannitolilytica*	respiratory infections	cystic fibrosis patients	USA	[69]
Ralstonia sp.	osteomyelitis, meningitis	nosocomial	(review)	[18]
Tepidimonas arfidensis	bone marrow sample	leukaemia patient	Korea	[72]
Cupriavidus gilardii	fatal infection	child with aplastic anaemia	USA	[71]
Hafnia alvei	urinary tract infection	elderly immunocompromised patient	Iraq	[90]
Enterobacter sakazakii	sepsis and meningitis	neonates and infants	USA	[91]
Enterobacter sakazakii	meningitis, enterocolitis, sepsis	neonates	Ireland	[14]
Enterobacter sakazakii		Isolated in infant formula	South Africa	[13]
Enterobacter sakazakii		neonates and infants	(review)	[15]
Cronobacter spp.	able to escape immune system	outbreaks	(review)	[46]
Cronobacter sakazakii			(review)	[16]
Kocuria kristinae	acute cholecystitis	post laparoscopic cholecystectomy	Hong Kong	[61]
Kocuria spp.			(review)	[60]
Kocuria kristinae	catheter-related bacteraemia and endocarditis	pulmonary septic emboli in pregnant female	USA	[89]
Kocuria spp.			(review)	[19]
Legionella pneumophila	pneumonia or Pontiac fever	diabetic male at hot springs facility	Japan	[10]
Arthrobacter scleromae	from swollen scleromata	dermatosis in a patient	China	[99]

Table S2. Closest match of 16S rDNA gene sequence from hot-spring isolates with GenBank and EzTaxon-e with percentage similarity and associated accession numbers.

	Genbank BLAST	ACCESSION NO	Ez taxon-e	ACCESSION NO	PHYLUM	CLASS	FAMILY	GENUS	SPECIES	SUBMITTED ACCESSION NO
57T	Kocuria sp 93%	HQ323439.1	Kocuria turfanensis 99.91%	DQ531634	Actinobacteria	Actinobacteria	Micrococcaceae	Kocuria	Kocuria turfanensis	MF120234
58T	Arthrobacter luteolus sp 99%	DQ486130.1	Arthrobacter luteolus 99.56%	AJ243422	Actinobacteria	Actinobacteria	Micrococcaceae	Arthobacter	Arthrobacter luteolus	MF120235
61T	uncultured Azospirillum 93%	HG917273.1	Skermanella aerolata 92.14%	DQ672568	Proteobacteria	Alpha proteobacteria	Rhodospirillaceae			MF120236
87T	Sphingomonas echinoides 99%	KP208156.1	Sphingomonas echinoides 99.9	JH584237	Proteobacteria	Alpha proteobacteria	Sphingomonadaceae	Sphingomonas	Sphingomonas echinoides	MF120239
5T	beta Proteabacterium 99%	KM054705.1	Zoogloea 98.14%	U46748	Proteobacteria	Beta proteobacteria	Rhodocyclaceae	Zoogloea		MF120227
27M	Gulbenkiania mobilis 99%	NR_042548.1	Gulbenkiania mobilis 99.93%	AM295491	Proteobacteria	Beta proteobacteria	Neisseriaceae	Gulbenkiania	Gulbenkiania mobilis	MF120228
31Lk	Burkholderia sp 99%	AY005039.1	Cupriavidus gilardii 99.52%	EU024163	Proteobacteria	Beta proteobacteria	Burkholderiaceae	Cupriavidus	Cupriavidus gilardii	MF120229
37Lb	Tepidimonas sp 99%	KF206375.1	Tepidimonas fonticaldi 98.01%	JN713899	Proteobacteria	Beta proteobacteria	Unclassified Burkholderiales	Tepidimonas		MF120230
42T	uncultured Tepidimonas 99%	HF912299.1	Tepidimonas fonticaldi 99.81	JN713899	Proteobacteria	Beta proteobacteria	Unclassified Burkholderiales	Tepidimonas	Tepidimonas fonticaldi	MF120231
55M	uncultured tepidimonas 92%	HF912299.1	Tepidimonas fonticaldi 92.43%	JN713899	Proteobacteria	Beta proteobacteria	Unclassified Burkholderiales			MF120232
59Lk	uncultured Aquabacterium 90%	KF598759.1	Aquabacterium fontiphilum 90.51%	EF626687	Proteobacteria	Beta proteobacteria	Unclassified Burkholderiales			MF120233
69Lk	Ralstonia 97%	CP010799.2	Ralstonia mannitolilytica 99.26%	AJ270258	Proteobacteria	Beta proteobacteria	Burkholderiaceae	Ralstonia	Ralstonia mannitolilytica	MF120237
44M	Silanimonas sp 99%	KF206368.1	Silanimonas lenta 92.52%	AUBD01000017	Proteobacteria	Gamma proteobacteria	Xanthomonadaceae	Silanimonas		MF144571

72T	uncultured bacterium 93%	AY959011.1	Cronobacter sakazakii 89.64%	BAWU01000071	Proteobacteria	Gamma proteobacteria	Enterobacteriaceae			MF144572
79M	Hafnia alvei 99%	KC210872.1	Hafia paralvei 99.43%	FM179943	Proteobacteria	Gamma proteobacteria	Enterobacteriaceae	Hafnia	Hafnia alvei	MF120238
80Lk	Cronobacter sakazakii 95%	HQ880369.1	Cronobacter dublinensis 91.38%	EF059892	Proteobacteria	Gamma proteobacteria	Enterobacteriaceae	Cronobacter		MF144573

Table S3. List of the accession numbers of all the strains from GenBank used in phylogenetic analysis.

BACTERIAL ISOLATE	NO. OF BASES	GENBANK ACCESSION NO
Arthrobacter luteolus strain CF-25	1499	ACCESSION NR_025362
Arthrobacter aurescens strain TC1; ATCC BAA-1386	1481	NCBI Reference Sequence: NR_074272.1
Uncultured *Arthrobacter* sp. clone TPB_GMAT_AC3.	1303	ACCESSION HG327131
Arthrobacter sp. GM37AC3 K2	1303	GenBank: HE798206.1
Arthrobacter sp. NCCP-1348	1465	ACCESSION LC065375
Arthrobacter woluwensis strain GQ-9	1432	ACCESSION KT072630
Arthrobacter woluwensis strain 1551	1477	ACCESSION NR_044894
Arthrobacter mysorens strain SBANHCu24	1385	ACCESSION KR152305
Arthrobacter mysorens strain DSM 12798	1491	ACCESSION NR_025613
Arthrobacter cumminsii strain DMMZ 445	1483	ACCESSION NR_044895
Arthrobacter cumminsii strain Z486	1414	GenBank: EU086827.1
Kocuria kristinae strain DSM 20032	1475	NCBI Reference Sequence: NR_026199.1
Kocuria varians strain ATCC 15306	1333	NCBI Reference Sequence: NR_114674.1
Kocuria marina	1434	GenBank: LC055502.1
Kocuria rhizophila strain TA68	1471	NCBI Reference Sequence: NR_026452.1
Kocuria himachalensis strain K07-05	1459	NCBI Reference Sequence: NR_043323.1
Kocuria polaris strain CMS 76or	1480	ACCESSION NR_028924
Kocuria rosea strain DSM 20447	1481	ACCESSION NR_044871
Kocuria aegyptia strain YIM 70003	1492	ACCESSION NR_043511
Kocuria sediminis strain FCS-11	1429	ACCESSION NR_118222
Kocuria flava strain HO-9041	1443	ACCESSION NR_044308
Kocuria sp. B38	1309	ACCESSION KC492107
Kocuria turfanensis strain HO-9042	1441	ACCESSION NR_043899
Uncultured methanogenic archaeon clone 6	1439	GenBank: DQ372975.1
Cupriavidus metallidurans strain CH34	1493	NCBI Reference Sequence: NR_027607.1
Cupriavidus gilardii strain CIP 105966	1315	NCBI Reference Sequence: NR_116146.1
Cupriavidus gilardii strain LMG 5886	1451	NCBI Reference Sequence: NR_114460.1
Cupriavidus sp. NCCP-1142	1557	ACCESSION LC065169
Gulbenkiania mobilis strain V28	1404	ACCESSION KC492099
Gulbenkiania mobilis E4FC31T	1498	ACCESSION AM295491
Ralstonia pickettii strain ATCC 27511	1491	NCBI Reference Sequence: NR_043152.1
Ralstonia syzygii ATCC 49543	1513	GenBank: AB021403.1
Ralstonia mannitolilytica strain LMG6866	1449	NCBI Reference Sequence: NR_025385.1
Tepidimonas sp. BR5	1257	GenBank: KF206377.1
Tepidimonas fonticaldi strain AT-A2	1457	NCBI Reference Sequence: NR_109514.1
Tepidimonas arfidensis	1442	AC AB206468;
Uncultured *Tepidimonas* sp. clone TPB_GMAT_5_1	1494	ACCESSION HE575486
Hafnia paralvei strain ATCC 29927	1495	NCBI Reference Sequence: NR_116898.1
Hafnia alvei strain ATCC 13337	1448	NCBI Reference Sequence: NR_044729.2
Cronobacter sakazakii strain ATCC 29544	1438	NCBI Reference Sequence: NR_118449.1
Cronobacter sakazakii ATCC BAA-894	1542	NCBI Reference Sequence: NR_102490.1
Silanimonas lenta strain 25-4	1382	NCBI Reference Sequence: NR_025815.1
Skermanella aerolata KACC 11604	1355	ACCESSION DQ672568
Sphingomonas echinoides strain ATCC 14820	1463	NCBI Reference Sequence: NR_024700.1
Sphingomonas paucimobilis strain DSM 30198	1462	NCBI Reference Sequence: NR_104893.1

Table S4. List of emerging opportunistic pathogens that have been isolated from hot-spring water.

Opportunistic pathogen	Country
Free-living amoeba	Taiwan [80]
Kocuria	Iran [39, 40]
Arthrobacter	Japan [39]
Sphingomonas	China [52]
Ralstonia	Korea [37]; Pakistan [37]
Gulbenkiania	India [73]
Tepidimonas	Taiwan [41]
Legionella	Thailand [11]; China [77]; Japan [79]; USA [79]; Poland [78]
Silanimonas	Korea [64]
Cronobacter	Malaysia [75]; Saudi Arabia [32]

List S1: Raw sequence data to be submitted to GenBank.

1. ***Zoogloea* sp. strain 5T 16S ribosomal DNA gene, partial sequence**

```
LOCUS       EMBOSS_001          642 bp    DNA     linear
ORIGIN
        1 CTCCTACGGG AGGCAGCAGT GGGGAATTTT GGACAATGGG CGCAAGCCTG ATCCAGCCAT
       61 GCCGCGTGAG TGAAGAAGGC CTTCGGGTTG TAAAGCTCTT TCAGTCGGGA AGAAAAGGCG
      121 GGCTCTAACA TAGCCTGTTG CTGACGGTAC CGACAGAAGA AGCACCGGCT AACTACGTGC
      181 CAGCAGCCGC GGTAATACGT AGGGTGCGAG CGTTAATCGG AATTACTGGG CGTAAAGCGT
      241 GCGCAGGCGG TTTGCTAAGA CAGGTGTGAA ATCCCCGGGC TTAACCTGGG AACTGCGCTT
      301 GTGACTGGCA GGCTAGAGTA CGGCAGAGGG GGGTGGAATT CCACGTGTAG CAGTGAAATG
      361 CGTAGAGATG TGGAGGAACA CCGATGGCGA AGGCAGCCCC CTGGGCCTG TACTGACGCT
      421 CATGCACGAA AGCGTGGGGA GCAAACAGGA TTAGATACCC TGGTAGTCCA CGCCCTAAAC
      481 GATGTCAACT AGTCGTTCGG AGCGGTAACG CACTGAGTGA CGCAGCTAAC GCGTGAAGTT
      541 GACCGCCTGG GGAGTACGGC CGCAAGGTTA AAACTCAAAG GAATTGACGG GGACCCGCAC
      601 AAGCGGTGGA TGATGTGGAT TAATTCGATG CAACGCGAAA AA
//
```

2. ***Gulbenkiania mobilis* strain 27M 16S ribosomal DNA gene, partial sequence**>27M

```
LOCUS       EMBOSS_001          640 bp    DNA     linear
ORIGIN
        1 CTCCTACGGG AGGCAGCAGT GGGGAATTTT GGACAATGGG CGCAAGCCTG ATCCAGCCAT
       61 GCCGCGTGTC TGAAGAAGGC CTTCGGGTTG TAAAGGACTT TTGTCGGGGA GGAAATCCTC
      121 TGGGTTAATA CCCTAGGGGG ATGACAGTAC CTGAAGAATA AGCACCGGCT AACTACGTGC
      181 CAGCAGCCGC GGTAATACGT AGGGTGCAAG CGTTAATCGG AATTACTGGG CGTAAAGCGT
      241 GCGCAGGCGG TTGTGCAAGT CTGATGTGAA ATCCCCGGGC TTAACCTGGG AACTGCATTG
      301 GAGACTGCAC GGCTAGAGTG CGTCAGAGGG GGGTGGAATT CCGCGTGTAG CAGTGAAATG
      361 CGTAGAGATG CGGAGGAACA CCGATGGCGA AGGCAGCCCC CTGGGATGAC ACTGACGCTC
      421 ATGCACGAAA GCGTGGGGAG CAAACAGGAT TAGATACCCT GGTAGTCCAC GCCCTAAACG
      481 ATGTCAACTA GCTGTTGGGG GGTTTGAATC CTTGGTAGCG TAGCTAACGC GTGAAGTTGA
      541 CCGCCTGGGG AGTACGGCCG CAAGGTTAAA ACTCAAAGGA ATTGACGGGG ACCCGCACAA
      601 GCGGTGGATG ATGTGGATTA ATTCGATGCA ACGCGAAGAA
//
```

3. ***Cupriavidus gilardii* strain 31Lk 16S ribosomal DNA gene, partial sequence**

LOCUS EMBOSS_001 602 bp DNA linear
ORIGIN

```
      1  GGACACCCTG CATCAGCATG CCGCGTGTGT GAAGAAGGCC TTCGGGTTGT AAAGCACTTT
     61  TGTCCGGAAA GAAATCGCGC TGGCTAATAC CTGGCGTGGA TGACGGTACC GGAAGAATAA
    121  GCACCGGCTA ACTACGTGCC AGCAGCCGCG GTAATACGTA GGGTGCGAGC GTTAATCGGA
    181  ATTACTGGGC GTAAAGCGTG CGCAGGCGGT TTTGTAAGAC AGGCGTGAAA TCCCCGGGCT
    241  TAACCTGGGA ATGGCGCTTG TGACTGCAAG GCTAGAGTGC CGTCAGAGGG GGGTAGAATT
    301  CCACGTGTAG CAGTGAAATG CGTAGAGATG TGGAGGAATA CCGATGGCGA AGGCAAGCCC
    361  CCTGGGACGA GACTGACGCT CATGCACGAA AGCGTGGGGA GCAAACAGGA TTAGATACCC
    421  TGGTAGTCCA CGCCCTAAAC GATGTCAACT AGTTGTTGGG GGATTCATTT CCTCAGTAAC
    481  GTAGCTAACG CGTGAAGTTG ACCGCCTGGG GAGTACGGTC GCAAGATTTA AAACTCAAAG
    541  GAATTGACGG GGACCCGCAC AAGCGGTGGA TGATGTGGAT TAATTCGATG CAACGCGAAA
    601  AA
```

//

4. *Tepidimonas* sp. strain 37Lb 16S ribosomal DNA gene, partial sequence>37LB

LOCUS EMBOSS_001 588 bp DNA linear
ORIGIN

```
      1  CCACCATGCC GCGTGCGGGG AAGAAGCCTT CGGGTTGTAA CCGCTTTTGT ACGGAACGAA
     61  AGGCTCTGGC TAATACCTGG GGCTCATGAC GGTACCGTAA GAATAAGCAC CGGCTAACTA
    121  CGTGCCAGCA GCCGCGGTAA TACGTAGGGT GCGAGCGTTA ATCGGAATTA CTGGGCGTAA
    181  AGCGTGCGCA GGCGGTTGTG TAAGACAGGC GTGAAATCCC CGGGCTCAAC CTGGGAAATG
    241  GCGCTTGTGA CTGCACGGCT GGAGTGCGGC AGAGGGGGGA TGGAATTTCC GCGTGTAGCA
    301  GTGAAATGCG TAGATATGCG GAGGAACACC GATGGCGAAG GCAGTCCCCT GGGCCTGCAC
    361  TGACGCTCAT GCACGAAAAG CGTGGGGAGC AAACAGGATT AGATACCCTG GTAGTCCACG
    421  CCCTAAACGA TGTCGACTGG TTGTTGGGCC TTAGGTGGCT CAGTAACGAA GCTAACGCGT
    481  GAAGTCGACC GCCTGGGGAG TACGGCCGCA AGGTTGAAAC TCAAAGGAAT TGACGGGGAC
    541  CCGCACAAGC GGTGGATGAT GTGGTTTAAT TCGATGCAAC GCGAAAAA
```

//

5. *Tepidimonas fonticaldi* strain 42T 16S ribosomal DNA gene, partial sequence

LOCUS EMBOSS_001 584 bp DNA linear
ORIGIN

```
      1  CCAGCATGCC GCGTGCGGGA GAGGCCTTCG GGTTGTAAAC CGCTTTTGTA CGGAACGAAA
     61  AGGCTCTGGC TAATACCTGG GGCTGATGAC GGTACCGTAA GAATAAGCAC CGGCTAACTA
    121  CGTGCCAGCA GCCGCGGTAA TACGTAGGGT GCGAGCGTTA ATCGGAATTA CTGGGCGTAA
    181  AGCGTGCGCA GGCGGTCTTG TAAGACAGAG GTGAAATCCC TGGGCTCAAC CTAGGAATGG
    241  CCTTTGTGAC TGCAAGGCTG GAGTGCGGCA GAGGGGGATG GAATTCCGCG TGTAGCAGTG
    301  AAATGCGTAG ATATGCGGAG GAACACCGAT GGCGAAGGCA GTCCCCTGGG CCTGCACTGA
    361  CGCTCATGCA CGAAAGCGTG GGGAGCAAAC AGGATTAGAT ACCCTGGTAG TCCACGCCCT
    421  AAACGATGTC GACTGGTTGT TGGGCCTTAG GTGGCTCAGT AACGAAGCTA ACGCGTGAAG
    481  TCGACCGCCT GGGGAGTACG GCCGCAAGGT TGAAACTCAA AGGAATTGAC GGGGACCCGC
    541  ACAAGCGGTG GATGATGTGG TTTAATTCGA TGCAACGCGA AAAA
```

//

6. *Silanimonas* sp. strain 44M 16S ribosomal DNA gene, partial sequence

LOCUS EMBOSS_001 647 bp DNA linear
ORIGIN

```
      1  CTCCTACGGG AGGCAGCAGT AGGGAATCTT CCGCAATGGA CGAAAGTCTG ACGGAGCAAC
     61  GCCGCGTGAG TGATGAAGGT CTTCGGGTTG TAAAGCTCTT TTGTTCCGGA AAAACAAGTC
    121  CCTGGATCTA ATACCTTCGG TGGGTTGACG GTACCGGACC AATAAGCACC GGCTAACTTC
    181  GTGCCAGCAG CCGCGGTAAT ACGAAGGGTG CAAGCGTTAC TCGGAATTAC TGGGCGTAAA
    241  GCGTGCGTAG GCGGTTCGTT AAGTCTGATG TGAAATCCCC GGGCTCAACC TGGGACGTGC
    301  ATTGGAAACT GGCGATCTAG AGTACGGTAG AGGGTGGTGG AATTCCCGGT GTAGCAGTGA
```

361 AATGCGTAGA GATCGGGAGG AACACTCGTG GCGAAGGCGG CCACCTGGAC CAGTAACTGA
421 CGCTGAGGCA CGAAAGCGTG GGGAGCCAAA CAGGATTAGA TACCCTGGTA GTCCACGCCC
481 TAAACGATGC GAACTGGATG TTGGGCTCAC TTCGGAGCTC AGTATCGAAG CTAACGCGTT
541 AAGTTCGCCG CCTGGGGAGT ACGGTCGCAA GACTGAAACT CAAAGGAATT GACGGGGGCC
601 CGCACAAGCG GTGGAGTATG TGGTTTAATT CGATGCAACG CGCAGAA
//

7. Betaproteobacteria strain 55M 16S ribosomal DNA gene, partial sequence

LOCUS EMBOSS_001 642 bp DNA linear
ORIGIN
1 CCTACGGGAG GCAGCAGTGG GGAATTTTGG ACAATGGGCG CAAGCCTGAT CCAGCAATGC
61 CGCGTGCGGG AAGAAGGCCT TCGGGTTGTA AACCGCTTTT GTACGGAACG AAAAGGCTCT
121 GGCTAATACC TGGGGCTGAT GACGGTACCG TAAGAATAAG CACCGGCTAA CTACGTGCCA
181 GCAGCCGCGG TAATACGTAG GGTGCGAGCG TTAATCGGAA TTACTGGGCG TAAAGCGTGC
241 GCAGGCGGTC TTGTAAGACA GAGGTGAAAT CCCTGGGCTC AACCTAGGAA TGGCCTTTGT
301 GACTGCAAGG CTGGAGTGCG GCAGAGGGGG ATGGAATTCC GCGTGTAGCG GTGAAATGCG
361 TAGAGATGCG GAGGAATACC GATGGCGAAG GCAGTCCCCT GGGCCGGCGA CTGACGCTCA
421 GGTACGAAAG CGTGGGGAGC AAAACAGGAT TAGATACCCT GGTAGTCCAC GCCCTAAACG
481 ATGTCGACTG GGAGGTTGGG CCCTTGATGG CGTCGCTTCC GGAGCTAACG CGTTAAGTCG
541 ACCGCCCTGG GGAGTACGGC CGCAAGGTTA AAACTCAAAT GAATTGACGG GGGCCCGCAC
601 AAGCGGTGGA GCATGTGGTT TAATTCGATG CAACGCGAAG AA
//

8. Betaproteobacteria strain 59Lk 16S ribosomal DNA gene, partial sequence

LOCUS EMBOSS_001 582 bp DNA linear
ORIGIN
1 GTTCCAGCGA TGCCGCGTGC AGGAGAGGTC TTCGGATTGT AACTGCTTTT GTCAGGAAAA
61 ATCTTCTGGG CTAATACCCC GGGAGGATAA TAGTACCTGA AGAATAATCC CCGGCTAACT
121 ACGTGCCAAC ACCCACGGTG CTACCTATGG TGCGAGCCTT AGTCCGAATT ACTGGTGTAA
181 CGCGTGCTCA GTCGTTATTT AAAAAAGATG TGACATCCCC GGGCTCAACC TGGAAACTGC
241 ATTTCTGACT GCATGACTGG ATGCTGCAAA GGGGGATGGA ATTCCTCGTG GAGCAGTGAA
301 ATGAGTAGAT ATGCGCAGGA ACACCGATGG CGAGGGCAAT CCCCTGGGCG TGCACTGACG
361 CTCATGCACG AAAGCGTGGG GAGCAAACAG GATTAGATAC CCTGGTAGTC CACGCCCTAA
421 TAGATGTCAA CTGGTTGTTG GACGGCTTGC TGTTCAGTAA CGAACCTAAC GCGTGAAGTT
481 GACCGCCTGG GGAGTACGGC CGCAAGGATG AAACTCAAAG GAATTGACGG GGACCCGCAC
541 GAGCGGTGGA TGATGTGGTT TAATTCGATG CTACGCGGAC AA
//

9. *Kocuria turfanensis* strain 57T 16S ribosomal DNA gene, partial sequence

LOCUS EMBOSS_001 995 bp DNA linear
ORIGIN
1 TTCGGATCGT AAACCTCTTT TGGCAGGGAA GAACAAGTAC CGTTCGAATA TGGCGGTACC
61 TTGACGGTAC CTAACCAGAA AGCGCCGGCT AACTACGTGC CAGCAGCCGC GGTAATACGT
121 AGGGCGCAAG CGTTGTCCGG AATTATTGGG CGTAAAGAGC TCGTAGGCGG TTTGTCGCGT
181 CTGCTGTGAA AGCCCGGGGC TCAACCCCGG GTCTGCAGTG GGTACGGGCA GAACTAGAGT
241 GCAGTTAGAG GGAGACTGGA ATTCCTGGTG TAGCGGTGAA ATGCGCAGAT ATCAGGAGGA
301 ACACCGATGG CGAAGGCAGG TCTCTGGGCT GTTACTGACG CTGAGGAGCG AAAGCATGGG
361 GGAGCGAACA GGGATTAGAT ACCCTGGGTA GTCCATGCCC GTAAACGTTG GGCACTAGGT
421 GTGGGGGACA TTTCCACGTT CTCCGCGCCG TAGCTAACGC ATTAAGTGCC CCGCCTGGGG
481 AGTACGGCCG CAAGGCTAAA ACTCAAAGGA ATTGACGGGG GCCCGCACAA GCGGCGGAGC
541 ATGCGGATTA ATTTCGATGC AACGCGAAAG AACCTTACCA AGGCTTGACA TTCACCGGAC
601 TACCTCAGAG ATGGGGTTTC CCTTCGGGGT CGGTGGACAG GTGGTGGCAT GGGTTGTCGT
661 CAGCTCGTGT CGTGAGATGT TGGGTTAAGT CCCGCAACGA GCGCAACCCT CGTTCTATGT

721 TGCCAGCACG TGATGGTGGG GACTCATAGG AGACTGCCGG GGTCAACTCG GAGGAAGGTG
781 GGGGATGACG TCAAATCATC ATGCCCCTTA TGTCTTGGGC TTCACGCATG CTACAATGGC
841 CGGTACAAAG GGTTGCGATA CTGTGAGGTG GAGCTAATCC CAAAAAGCCG GTCTCAGTTC
901 GGATTGAGGT CTGCAACTCG ACCTCATGAA GTCGGAGTCG CTAGTAATCG CAGATCAGCA
961 ACGCTGCGGT GAATACGTTC CCGGGCCTTG TACAC
//

10. *Arthrobacter luteolus* strain 58T 16S ribosomal DNA gene, partial sequence

LOCUS EMBOSS_001 958 bp DNA linear
ORIGIN
1 TTCGGGTTGT AAACCTCTTT CAGCAGGGAA GAAGCGAAAG TGACGGTACC TGCAGAAGAA
61 GCGCCGGCTA ACTACGTGCC AGCAGCCGCG GTAATACGTA GGGCGCAAGC GTTATCCGGA
121 ATTATTGGGC GTAAAGAGCT CGTAGGCGGT TTGTCGCGTC TGCTGTGAAA GCCCGGGGCT
181 CAACCCCGGG TCTGCAGTGG GTACGGGCAG ACTAGAGTGC AGTAGGGGAG ACTGGAATTC
241 CTGGTGTAGC GGTGAAATGC GCAGATATCA GGAGGAACAC CGATGGCGAA GGCAGGTCTC
301 TGGGCTGTAA CTGACGCTGA GGAGCGAAAG CATGGGGAGC GAACAGGATT AGATACCCTG
361 GTAGTCCATG CCGTAAACGT GGGCACTAGG TGTGGGGGAC ATTCCACGTT TTCCGCGCCG
421 TAGCTAACGC ATTAAGTGCC CCGCCTGGGG AGTACGGCCG CAAGGCTAAA ACTCAAAGGA
481 ATTGACGGGG GCCCGCACAA GCGGCGGAGC ATGCGGATTA ATTCGATGCA ACGCGAAGAA
541 CCTTACCAAG GCTTGACATG AACCGGTAAG ACCTGGAAAC AGGTCCCCCA CTTGTGGCCG
601 GTTTACAGGT GGTGCATGGT GTCGTCAGCT CGTGTCGTGA GATGTTGGGT TAAGTCCCGC
661 AACGAGCGCA ACCCTCGTTC TATGTTGCCA GCGGGTTATG CCGGGGACTC ATAGGAGACT
721 GCCGGGGTCA ACTCGGAGGA AGGTGGGGAC GACGTCAAAT CATCATGCCC CTTATGTCTT
781 GGGCTTCACG CATGCTACAA TGGCCGGTAC AAAGGGTTGC GATACTGTGA GGTGGAGCTA
841 ATCCCAAAAA GCCGGTCTCA GTTCGGATTG AGGTCTGCAA CTCGACCTCA TGAAGTGGAG
901 TCGCTAGTAA TCGCAGATCA GCAACGCTGC GGTGAATACG TTCCCGGGCC TTGTACAC
//

11. Alphaproteobacteria strain 61T 16S ribosomal DNA gene, partial sequence

LOCUS EMBOSS_001 541 bp DNA linear
ORIGIN
1 GCCTTCGGGT TGTAAAGCTC TTTCGCACGC GACGATGATG ACGGTAGCGT GAGAAGAAGC
61 CCCGGCTAAC TTCGTGCCAG CAGCCGCGGT AATACGAAGG GGGCTAGCGT TGTTCGGAAT
121 TACTGGGCGT AAAGGGCGCG TAGGCGGTGT GTCAAGTCAG GCGTGAAAGC CCCGGGCTCA
181 ACCTGGGAAC AGCGCTTGAG ACTGGCACGC TCGAGTTCGG GAGAGGATGG TGGAATTCCC
241 AGTGTAGAGG TGAATTCGTA GATATTGGGA AGAACACCCG ATGGCGAATG CAGCCATCTG
301 GACCGACACT GACGCTGAGG CGCGAAAAGC CGTGGGGAGC AAACAGGATT AGATACCCTG
361 GTAGTCCCCG CCCGTAAACG ATGAGTGCTA AACGTCGGGG CCCTTTAGGG TTTCGGTGTC
421 CCACTAACGC ATTAAGCACT CCCCCTGGGG GAGTCGGGCC GCAAGGTTAA AATCAAAGGA
481 ATTGACGGGG GCCCGCACAA GCGGTGGAAC ATGGCGGTTT AATTCGAAGG AACGAGCAGA
541 A
//

12. *Ralstonia mannitolilytica* strain 69Lk 16S ribosomal DNA gene, partial sequence

LOCUS EMBOSS_001 645 bp DNA linear
ORIGIN
1 CTCCTACGGG AGGCAGCAGT GGGGAATTTT GGACAATGGG CGAAAGCCTG ATCCAGCAAT
61 GCCGCGTGTG TGAAGAAGGC CTTCGGGTTG TAAAGCACTT TTGTCCGGAA AGAAATGGCT
121 CTGGTTAATA CCCGGAGTCG ATGACGGTAC CGGAAGAATA AGGACCGGCT AACTACGTGC

181 CAGCAGCCGC GGTAATACGT AGGGGTCCAA GCGTTAATCG GAATTACTGG GCGTAAAGCG
241 TGCGCAGGCG GTTGTGCAAG ACCGATGTGA AATCCCCCGG GCTTAACCTG GGAATTGCAT
301 TGGTGACTGC ACGGCTAGAG TGTGTCAGAG GGAGGTAGAA TTCCACGTGT AGCATGAAAT
361 GCGTAGAGAT GTGGAGGAAT ACCGATGGCG AAGCAGCCTC CCTGGGATAA CACTGACGCT
421 CATGCACGAA AAGCGTGGGG AGCAAACAGG ATTAGATACC CCTGGTAGTC CACGCCCCTA
481 AACGATGTCA ACTAAGTTGT TGGGGATTCA TTTCCTTAGT AACGTAGCTA ACGCGTGAAG
541 TTGACCGCCT GGGGAGTACG GTCGCAAGAT TAAAACTCAA AGGAATTGAC GGGGAACCCG
601 CACAAGCGGT GGATGATGTG GATTAATTCG ATGCAACGCG AAAAA
//

13. Enterobacteriaceae strain 72T 16S ribosomal DNA gene, partial sequence

LOCUS EMBOSS_001 638 bp DNA linear
ORIGIN
1 CCTACGGGAG GCAGCAGTGG GGAATATTGC ACAATGGGCG CAAGCCTGAT GCAGCCATGC
61 CGCGTGTATG AAGAAGGCCT TCGGGTTGTA AAGTACTTTC AGCGGGGAGG AAGGTGTTGT
121 GGTTAATAAC CGCAGCAATT GACGTTACCC GCAGAAGAAG CACCGGCTAA CTCCGTGCCA
181 GCAGCCGCGG TAATACGGAG GGTGCAAGCG TTAATCGGAA TTACTGGGCG TAAAGCGCAC
241 GCAGGCGGTT CTTAAGTCAG ATGTGAAATC CCCGGGCTCA ACCTGGGAAC TGCATTTGAA
301 ACTGGGAAGC TTGAGTCTCG TAGAGGGGGG TAGAATTCCA GGTGTAGCGG TGAAATGCGT
361 AGAGATCTGG AGGAATACCG GTGGCGAAGG CGACCCCCTG GTCTGAAACT GACGCTCAGG
421 CGCGAAAGCG TGGGGAGCGA ACAGGATTAG ATACCCTGGT AGTCCACGCC GTAAACGATG
481 AGTGCTAAGT GTTAGAGGCC TTCCGCCCTT TAGTGCTGCA GCAAACGCAT TAAGCACTCC
541 GCCTGGGGAG TACGGTCGCA AGACTGAAAC TCAAAGGAAT TGACGGGGGC CCGCACAAGC
601 GGTGGAGCAT GTGGTTTAAT TCGAAGCAAC GCGAAGAC
//

14. *Hafnia alvei* strain 79M 16S ribosomal DNA gene, partial sequence

LOCUS EMBOSS_001 583 bp DNA linear
ORIGIN
1 CCTTGCCGCG TGTATGAAAA GGCCTTCGGG TTGTAAAGTA CTTTCAGCGA GGAGGAAGGC
61 ATTAAGGTTA ATAACCTTAG TGATTGACGT TACTCGCAGA AGAAGCACCG GCTAACTCCG
121 TGCCAGCAGC CGCGGTAATA CGGAGGGTGC AAGCGTTAAT CGGAATTACT GGGCGTAAAG
181 CGCACGCAGG CGGTTGGTTA AGTCAGATGT GAAATCCCCG AGCTTAACTT GGGAACTGCA
241 TTTGAAACTG GCCAGCTAGA GTCTTGTAGA GGGGGGTAGA ATTCCAGGTG TAGCGGTGAA
301 ATGCGTAGAG ATCTGGAGGA ATACCGGTGG CGAAGGCGGC CCCCTGGACA AAGACTGACG
361 CTCAGGTGCG AAAGCGTGGG GAGCAAACAG GATTAGATAC CCTGGTAGTC CACGCTGTAA
421 ACGATGTCGA CTTGGAGGTT GTGCCCTTGA GGCGTGGCTT CCGGAGCTAA CGCGTTAAGT
481 CGACCGCCTG GGGAGTACGG CCGCAAGGTT AAAACTCAAA TGAATTGACG GGGGCCCGCA
541 CAAGCGGTGG AGCATGTGGT TTAATTCGAT GCAACGCGAA GAA
//

15. *Cronobacter* strain 80Lk 16S ribosomal DNA gene, partial sequence

LOCUS EMBOSS_001 649 bp DNA linear
ORIGIN
1 CTCCTACGGG AGGCAGCAGT AGGGAATTTT CCACAATGGA CGAAAGTCTG ATGGAGCAAC
61 GCCGCGTGAA CGATGAAGGT CTTCGGATTG TAAAGTTCTG TTGTCGGGGA CGAACACGTA
121 CCGTTTAATA ACGGCGGCAC ATTGACGGTA CCCGCAGAAA AAGCCACGGC TAACTACGTG
181 CCAGCAGCCG CGGTAATACG GAGGGTGCAA GCGTTAATCG GAATTACTGG GCGTAAAGCG
241 CACGCAGGCG GTCTATGTAA GTCAGATGTG AAATCCCCGG GCTCAACCTG GGAACTGCAT
301 TTGAAACTGT GTAGCTTGAG TCTCGTAGAG GGGGGTAGAA TTCCAGGTGT AGCGGTGAAA
361 TGCGTAGAGA TCTGGAGGAA TACCGGTGGC GAAGGCGGCC CCCTGGACTG AAGACTGACG
421 CTCAGGTGCG AAAAGCGTGG GGGAGCAAAC AGGATTAGAT ACCCTGGTAG TCCACGCCGT

481 TAAACGATGT CGACTTGGAT GTTGGGCCCT TGGAGGCGTG GCTTCCGGAG CTAACGCGTT
541 AAGTCGACCG CCTGGGGGAG TACGGCCGCA AGGTTAAAAC TCAAATGAAT TGACGGGGGC
601 CCGCACAAAG CGGTGGAGCA TGTGGTTTAA TTCGATGCAA CGCGAAGAA
//

16. *Sphingomonas echinoides* strain 87T 16S ribosomal DNA gene, partial sequence

LOCUS EMBOSS_001 563 bp DNA linear
ORIGIN
1 CAGCAATGCC GCGTGAGTGA TGAAGGCCTT AGGGTTGTAA AGCTCTTTTA CCCGGGATGA
61 TAATGACAGT ACCGGGAGAA TAAGCTCCGG CTAACTCCGT GCCAGCAGCC GCGGTAATAC
121 GGAGGGAGCT AGCGTTATTC GGAATTACTG GGCGTAAAGC GCACGTAGGC GGCTTTGTAA
181 GTTAGAGGTG AAAGCCTGGA GCTCAACTCC AGAATTGCCT TTAAGACTGC ATCGCTTGAA
241 TCCAGGAGAG GTGGAGTGGA ATTCCGAGTG TAGAGGTGAA ATTCGTAGAT ATTCGGAAGA
301 ACACCAGTGG CGAAGGCGGC TCACTGGACT GGTATTGACG CTGAGGTGCG AAAGCGTGGG
361 GAGCAAACAG GATTAGATAC CCTGGTAGTC CACGCCGTAA ACGATGATAA CTAGCTGTCG
421 GGGCTCTTAG AGCTTCGGTG GCGCAGCTAA CGCATTAAGT TATCCGCCTG GGGAGTACGG
481 CCGCAAGGTT AAAACTCAAA TGAATTGACG GGGGCCTGCA CAAGCGGTGG AGCATGTGGT
541 TTAATTCGAA GCAACGCGCA GAA
//

Reference

99. Huang, S.; Hsu, B.; Wu, S.; Fan, C.; Shih, F.; Lin, Y.; Ji, D. Water quality parameters associated with prevalence of *Legionella* in hot spring facility water bodies. *Water Res.* **2010**, *44*, 4805–4811.
